# A self-adaptive deep learning method for automated eye laterality detection based on color fundus photography

**DOI:** 10.1371/journal.pone.0222025

**Published:** 2019-09-19

**Authors:** Chi Liu, Xiaotong Han, Zhixi Li, Jason Ha, Guankai Peng, Wei Meng, Mingguang He

**Affiliations:** 1 State Key Laboratory of Ophthalmology, Zhongshan Ophthalmic Center, Sun Yat-sen University, Guangzhou, China; 2 School of Computer Science, University of Technology, Sydney, Australia; 3 Faculty of Medicine, Nursing and Health Sciences, Monash University, Clayton, Australia; 4 Guangzhou Healgoo Interactive Medical Technology Co. Ltd., Guangzhou, China; 5 Centre for Eye Research Australia, Royal Victorian Eye and Ear Hospital, Melbourne, Australia; 6 Ophthalmology, Department of Surgery, University of Melbourne, Melbourne, Australia; Federal University of São Paulo, BRAZIL

## Abstract

**Purpose:**

To provide a self-adaptive deep learning (DL) method to automatically detect the eye laterality based on fundus images.

**Methods:**

A total of 18394 fundus images with real-world eye laterality labels were used for model development and internal validation. A separate dataset of 2000 fundus images with eye laterality labeled manually was used for external validation. A DL model was developed based on a fine-tuned Inception-V3 network with self-adaptive strategy. The area under receiver operator characteristic curve (AUC) with sensitivity and specificity and confusion matrix were applied to assess the model performance. The class activation map (CAM) was used for model visualization.

**Results:**

In the external validation (N = 2000, 50% labeled as left eye), the AUC of the DL model for overall eye laterality detection was 0.995 (95% CI, 0.993–0.997) with an accuracy of 99.13%. Specifically for left eye detection, the sensitivity was 99.00% (95% CI, 98.11%-99.49%) and the specificity was 99.10% (95% CI, 98.23%-99.56%). Nineteen images were wrongly classified as compared to the human labels: 12 were due to human wrong labelling, while 7 were due to poor image quality. The CAM showed that the region of interest for eye laterality detection was mainly the optic disc and surrounding areas.

**Conclusion:**

We proposed a self-adaptive DL method with a high performance in detecting eye laterality based on fundus images. Results of our findings were based on real world labels and thus had practical significance in clinical settings.

## Introduction

With the rapid development of artificial intelligence (AI)-based image identification and grading, fundus images have shown great promise in diagnosing and monitoring eye diseases including diabetic retinopathy, age-related macular degeneration and glaucoma.[1–3] However, most previous studies have focused solely on disease detection or grading for single fundus photography irrespective of eye laterality (left or right eye), which is one of the key pieces of information in fundus images and also a basic element for ophthalmic diagnosis. Thus automatic and accurate eye laterality detection must be addressed before wider application of AI-based diagnosis in ophthalmic clinical practice. Currently, the eye laterality of fundus image is recorded as a meta data information during fundus photography examination according to the pre-restricted examination sequence (i.e., first right eye, then left eye), or manually labeled after image acquisition. Both methods have limitations: the former suffering from infexibility while the latter is time-consuming and error-prone. In addition, correct eye labels are needed for multiple large open-access fundus image sets, such as Eye-PACS[4] and LabelMe[2], and new data banking projects in the future also call for an accurate and automatic way for eye laterality detection of color fundus photography.

Recent studies have demonstrated that deep learning (DL) has been applied in fundus image-based eye disease diagnosis and grading with great success.[5] However, very few studies exist investigating automatic eye laterality detection based on fundus photography and mostly rely on traditional feature engineering like pixel intensity detection[6] or local anatomical features extraction[7], which is easily affected by image quality and whether the region of interest (ROI), for instance optic disc or macula, is included in the image. Additionally, one major concern for most of the existing literature is that human subjective labels have been used as the ground truth to train the DL system, which brings to question its real-world reliability and practicability.[2] Another concern is referred to as the “black box problem”, which calls for a better understanding and demonstration of the DL system on how it arrives at its decisions.[8] Moreover, the current DL networks are mostly trained by fixed hyper-parameters selected after multiple trials, which can be improved with a self-adaptive method.[9, 10]

In this study, we proposed a self-adaptive DL method to automatically detect the eye laterality of fundus images based on real-world labels from one large epidemiology study, and further validated the method in an independent online dataset to assess its performance. In addition, we compared the performance of multiple image pre-processing methods and provided visualization to highlight the ROI for eye laterality detection.

## Methods

### Overview

**Fig 1:**
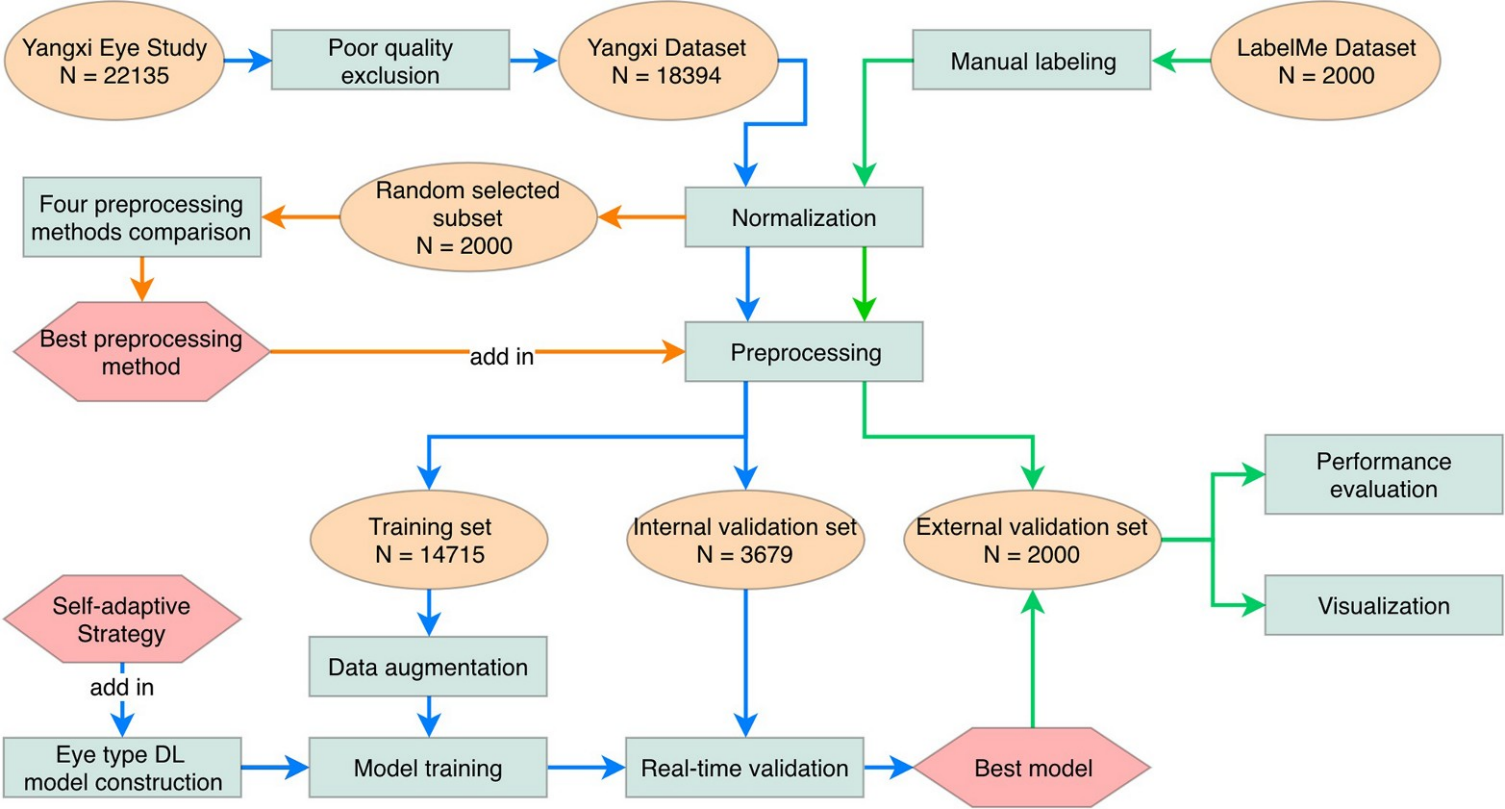
Integrated workflow in this study. The blue, green and orange color arrows show sub-workflows in preprocessing methods comparison, model training and external validation, respectively.

The proposed method consisted of four main steps. Firstly, all images were normalized into the same size. Then four image preprocessing methods were tested in a random subset of 2000 fundus images, and the method with the best performance was applied to the whole image set for the subsequent DL model training. Secondly, a self-adaptive DL model for automated eye laterality detection was trained and validated in 14715 and 3679 pre-processed images from the Yangxi Dataset, respectively. Thirdly, the trained DL model was further validated in 2000 pre-processed images from an independent LabelMe Dataset. Lastly, the visualization of the DL model was presented. Details of the individual steps were described below. Fig 1 illustrates the overall workflow of the proposed method in this study.

### Ethics statement

The current study was approved by the Zhongshan Ophthalmic Center Institutional Review Board (2017KYPJ049) and the tenets of the Declaration of Helsinki were observed. Given the retrospective nature and fully anonymized usage of images in this study, the review board waived the requirement for the informed consent of patients participated in this study.

### Details of image datasets

The Yangxi Eye Study is a population-based epidemiology study which enrolled 5825 adults aged 50 years or older in Yangxi, Guangdong.[11] Fundus photography was taken for the right eye first and then the left eye in each participant (at least one optic disc-centered/macula-centered image in each eye) by trained nurses using a non-mydriatic digital fundus camera (FundusVue, Taiwan). The eye laterality was labeled by the camera according to the pre-defined examination sequence and double-checked by the photographer on field. A total of 22135 fundus images were eventually obtained from the Yangxi Eye Study.

An exclusion criterion regarding image quality was imposed to avoid data noise for model training. Images in which ≥50% of the area was obscured or only part of optic disc was visible were defined as poor quality and excluded. After excluding poor quality images by three trained graders, a total of 18394 fundus images with real-world eye laterality labels were included in this study (referred to as the Yangxi Dataset), which was randomly divided into a training dataset of 14715 images and an internal validation dataset of 3679 images.

In addition, 2000 fundus images were randomly sampled and downloaded from the online database LabelMe (Healgoo Ltd. LabelMe Database; 2016. http://www.labelme.org. Accessed February 16, 2016.), which contains more than 320000 color fundus images collected using various fundus cameras, including Topcon, Canon, Heidelberg and Digital Retinography System, from various clinical settings in China. These 2000 images were used for external validation, with eye laterality labeled by one trained specialist. Table 1 shows the eye laterality distribution of the Yangxi and LabelMe Datasets. Note that the exclusion criterion was only applied in the Yangxi Dataset, but not the LabelMe Dataset due to that the model was expected to be evaluated in a real-world dataset without any human intervention for external validation.

**Table 1:** The eye laterality distribution of the Yangxi and LabelMe Datasets.

	Yangxi Dataset		LabelMe Dataset
	Training set	Internal validation set	External validation set
**Left eye**	7320	1831	1000
**Right eye**	7395	1848	1000
**In total**	14715	3679	2000

### Data preparation

#### Image size normalisation

Since various different fundus cameras were used during image acquisition, the original images in this study had 3 different sizes, 2560*1920, 3280*2480, 4700*3100 (S1 Fig). Before model training, all the original images were normalized into 3-channel RGB images, and a black edge of each image was cut off based on pixel summation to maintain square dimensions. Then all the images were rescaled to a size of 299*299 in consideration of the hardware’s computational capacity. Lastly, a circular mask with a radius equal to 95% of the fundus area radius was used to filter the overexposed edges to further extract the key fundus area (S2 Fig).

#### Comparison of four image preprocessing methods

**Fig 2:**
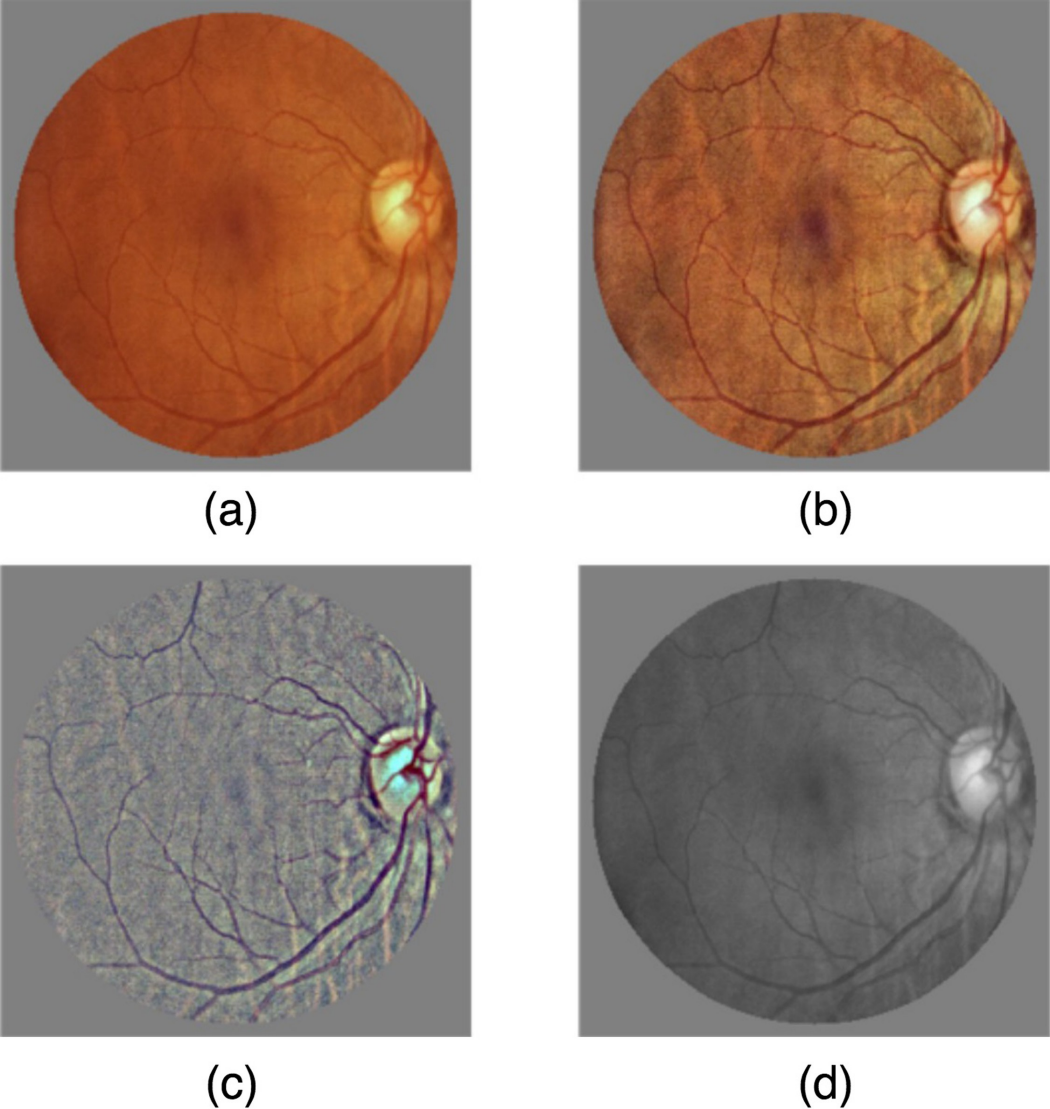
Demonstration of the four image preprocessing methods. (a) ORIGINAL; (b) CLAHE; (c) LSACR; (d) GRAY.

After image size normalization, image preprocessing methods were used to enhance the feature illustration on fundus images. Four image preprocessing methods were tested and the method with the best performance was used for subsequent model training (Fig 2):

- No preprocessing (referred as ORIGINAL): Directly input the original images.
- Contrast-limited Adaptive Histogram Equalization (CLAHE): Redistribute the pixels equally over the whole histogram to enhance the contrast, thereby emphasizing local features.[12]
- Local Space Average Color Removal (LSACR): Remove the local space average color by a Gaussian filter to obtain balanced color and illumination.[13, 14]
- Gray transformation (referred as GRAY): transform the RGB images into gray images.[15]

A random subset of 2000 images from the Yangxi Dataset were used for this analysis, which was preprocessed by the 4 methods respectively and randomly divided into a training set and a validation set with a proportion of 8:2. A DL model was trained, as further described below, during which a real-time accuracy-loss curve of the validation set was recorded to compare the performances of these 4 methods.

### Development of the self-adaptive deep learning model

#### Deep learning model architecture

Inception-V3 is one of the state-of-art DL models proposed by Christian et al., which uses various inception modules to improve the feature dimensional diversity.[16] Its effectiveness has been widely proved in fundus image-based automated eye disease diagnosis.[2, 3, 10] Our DL model was constructed based on a fine-tuned Inception-V3 network with a total of 11 inception modules (S3 Fig).

When inputted an image pixel vector with a size of (299,299,3), the model will correspondingly output a probability vector (*P*1, *P*2). Specifically, *P*1 referred to the probability of being detected as the left eye while *P*2 referred to the probability of being detected as the right eye. Stochastic Gradient Descent (SGD)[17] algorithm was used to update the network weights during the training phase. Data augmentation was performed to enlarge image heterogeneity while retaining prognostic features, which included a random horizontal shift of 0–10 pixels and a random rotation of 30 degree of the images. The cross-entropy function with L2 regularization was used as the loss function to decay the model weights.

#### Self-adaptive strategy

We proposed a self-adaptive strategy in the model training, which was able to automatically adjust the learning rate and select the current best model weights during training. Specifically, a monitor *M* was added to the model training, to allow the model to make automated adjustments to improve subsequent training when *M* reached a pre-defined threshold. In order to minimize the loss and maximum the accuracy simultaneously, we defined *M* as Eq (1),

M=(1−Accuracy)*Loss (1)

Evidently, a smaller *M* indicated a better performance.

- Self-adaptive learning rate
  At the beginning of model training, the learning rate *l* was initialized as a relatively bigger value *l*_0_ = 0.1. Then *M* was checked after each training epoch. Assuming the index value at the end of epoch *n* is *M*_*n*_, if *M*_*n*+1_ < *M*_*n*_, *l* remains unchanged. Otherwise if *M*_*n*+1_ ≥ *M*_*n*_, an alert period will start. Then in the following *k* epochs, if Eq (2) stands, *l* will automatically reduce following Eq (3)
  $$M_{n+i}\geq M_{n},\;i\in \left[1,2,..,k\right]$$ (2)
  $$l_{new}=l\text{*}\alpha ,\;0<\alpha \leq 1$$ (3)
  where *α* means the reduction factor. In this study we set *k* = 5 and *α* = 0.5.
- Self-adaptive best model selection
  A model pool was added to the training, which could save and update the current best model adaptively. Assuming the monitor of the current best model is *M*_*best*_, then the update rule of model pool is as follows.

At the end of epoch *n*, The model pool would update the current best model to the model generated after epoch *n*, if *M*_*n*_, *Accuracy* and *Loss* satisfy:

$$M_{n}\leq M_{best}\leq M_{threshold}$$

Accuracy≥0.95

Loss≤0.1 (4)

Where *M*_*threshold*_ was the pre-determined threshold of *M* and set as 0.005 in this study. The best model in the model pool would be automatically loaded into the current training process after every 10 epochs to avoid manual model selection.

The self-adaptive strategy was only used for the eye laterality detection model but not for the image preprocessing methods comparison model. All the models and strategies were implemented using Keras (version 2.1.4), an open-source Python library for DL.

### Evaluation and statistical analysis

During model training, the real-time *Accuracy*, *Loss* and *M* recorded after each epoch were used as the evaluation metrics and monitoring index. The best model was selected based on the final accuracy-loss curve and the model was tested in the internal and external validation datasets, respectively. A receiver operating characteristic (ROC) curve and confusion matrix were used for performance evaluation. Accuracy, sensitivity and specificity were also calculated. Given the eye laterality detection was a binary classification task, the ROC curve, accuracy, sensitivity and specificity were only reported for the detection of left eye.

### Visualization

To better understand and demonstrate the DL network, we used Class Activation Maps (CAM) proposed by Bolei et al. for model visualization in this study.[18] A weighted sum of the feature maps outputted by the last convolutional layer of the DL model was computed to obtain the CAM. Then a heat map was generated based on the CAM to highlight the ROI for the DL model in eye laterality detection.

## Results

### Comparison of four image preprocessing methods

**Fig 3:**
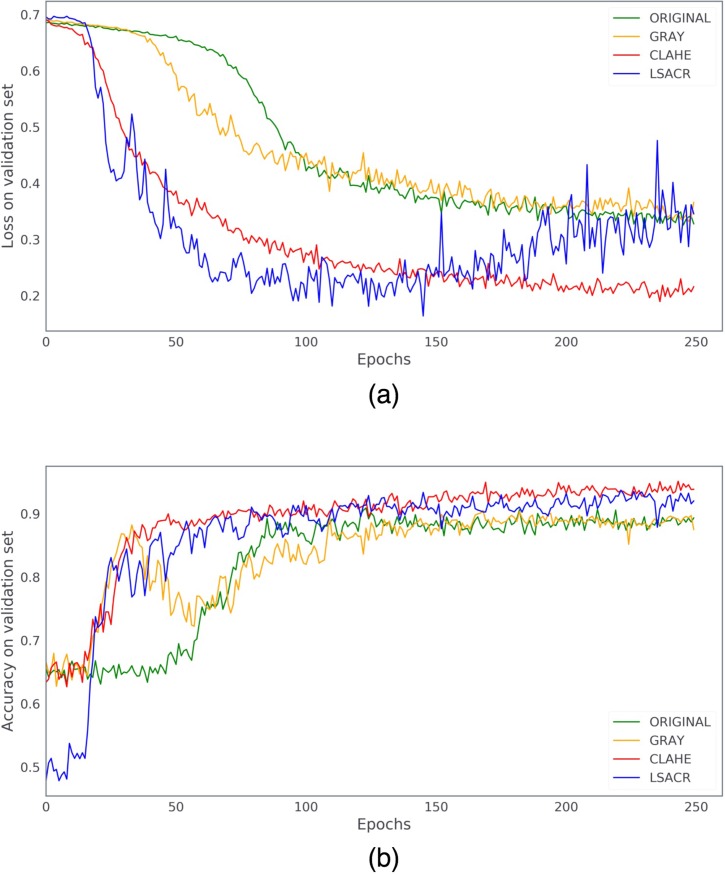
The real-time accuracy-loss curves of the 4 image preprocessing methods during 250 training epochs. (a) Loss in the validation set; (b) Accuracy in the validation set.

Fig 3 shows the real-time accuracy-loss curves of the 4 preprocessing methods recorded during training. It could be seen that, CLAHE and LSACR had overall much lower loss and higher accuracy in the validation set than GRAY and ORIGINAL, representing a better performance. Furthermore, CLAHE outperformed LSACR in that the loss curve of CLAHE was more smooth, and that overfitting started at around 150 epochs with LSACR but there was no sign of overfitting with CLAHE during the 250 epochs. In consideration of accuracy, we also found that CLAHE had better accuracy than LSACR after 150 epochs. Thus CLAHE was selected as the image preprocessing method for subsequent eye laterality detection model training.

### Self-adaptive deep learning model training

**Fig 4:**
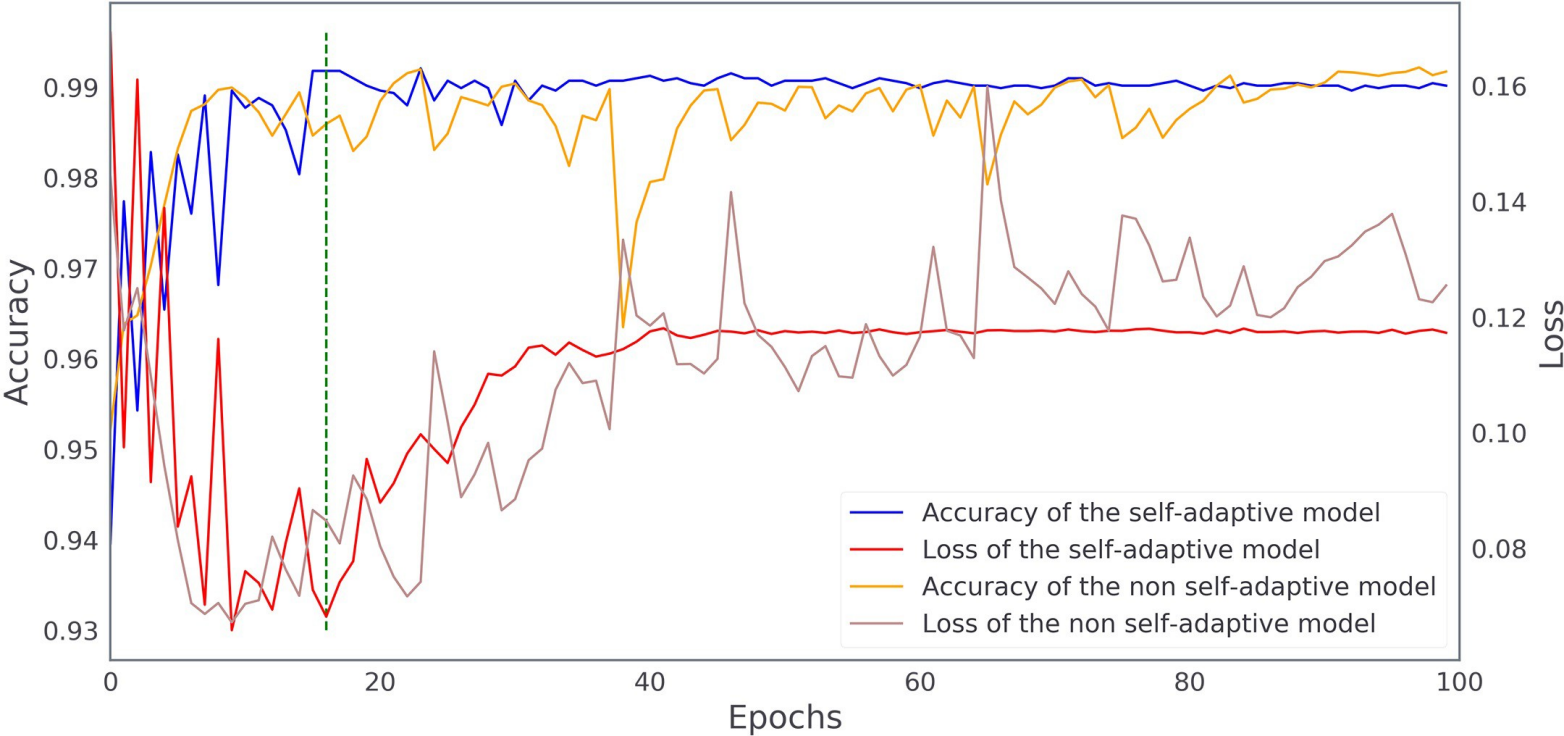
The real-time accuracy-loss curves of the deep learning model with and without self-adaptive strategy. The green dashed line indicates the epoch when the best model was selected.

Fig 4 shows the real-time accuracy-loss curves of the eye laterality detection model with and without the self-adaptive strategy, recorded in the internal validation set. As shown, the self-adaptive model had a more stable learning curve and an overall higher accuracy than the non self-adaptive model. In addition, overfitting started later for the self-adaptive model (at about epoch 16) compared with the non-adaptive model (at about epoch 10). The self-adaptive model at epoch 16 was selected as the final model for eye laterality detection and further tested in the internal and external validation datasets.

### Model performance evaluation

**Fig 5:**
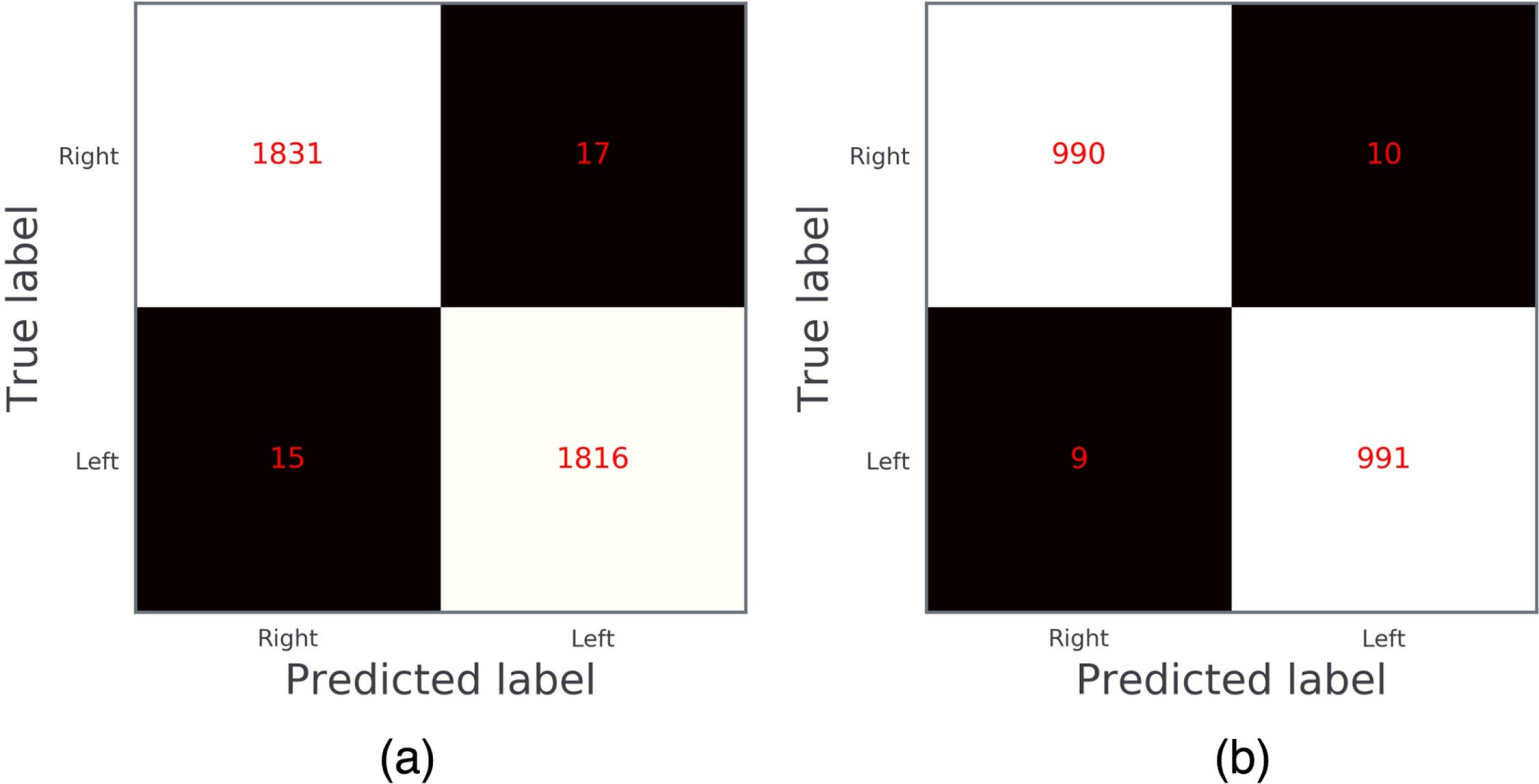
Confusion matrix of the deep learning model for eye laterality detection. (a) The internal validation; (b) The external validation.

For left eye detection, in the internal validation set (N = 3679 images, 49.77% left eye), The DL model had an accuracy of 99.13%, and the sensitivity was 99.18%, specificity was 99.08%; while in the external test dataset from LabelMe (N = 2000 images, 50% labeled left eye), the AUC was 0.9946 (95%CI, [0.9913–0.9974]) with an accuracy of 99.02%, the sensitivity was 99.10%, and the specificity was 99.00% (Figs 5 and S4).

Nineteen images from the external validation dataset were wrongly labeled by the DL model as compared to human labels, reasons included: 1) wrong human label in the LabelMe dataset (12 images); 2) images without major anatomical features (7 images, S5 Fig).

### Visualization result

**Fig 6:**
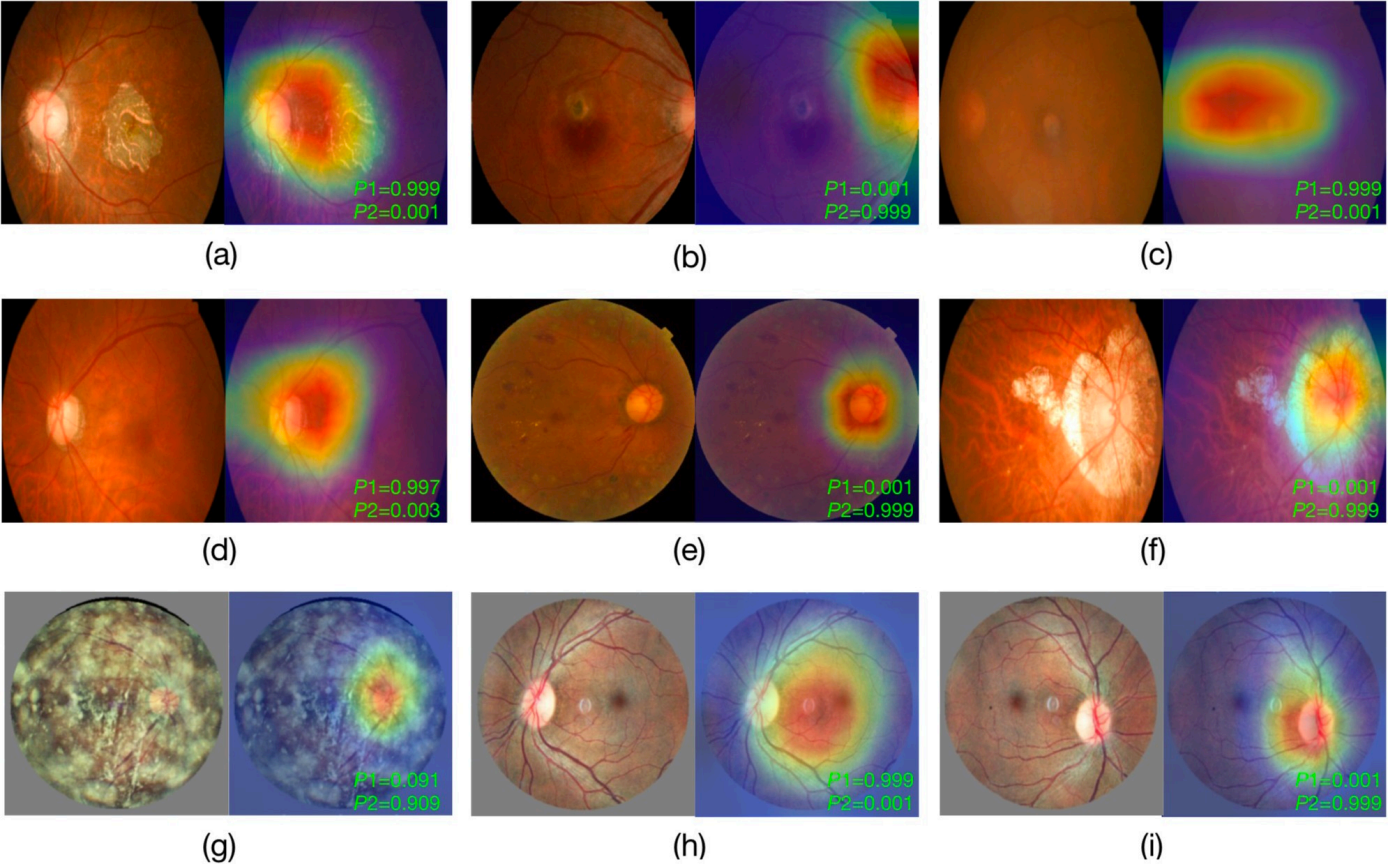
Visualization of the deep learning model on fundus images with different eye conditions. The eye conditions from (a) to (g) are: (a) late dry age-related macular degeneration (AMD); (b) late wet AMD with disc tilt; (c) cataract; (d) glaucoma; (e) diabetic retinopathy; (f) high myopia; (g) vitreous opacity. (h) and (i) are two fundus images from both eyes of the same person. The bottom-right green text in each example indicates the corresponding probability scores outputted by the model (*P*1: left eye; *P*2: right eye).

We performed CAM visualization on several randomly selected image samples, which highlighted the ROI of the DL model regarding eye laterality detection. Fig 6 demonstrates that for both disc-centered or macula-centered fundus images, the ROI was mainly focused on the optic disc and surrounding areas. In addition, as long as the image quality was acceptable (i.e., the disc and surrounding areas were clearly visible), the ROI kept invariant in images with varying eye conditions, even those with severe fundus pathologies. As shown in Fig 6, the model had significant confidence in making the correct decision, regardless of the eye condition.

Another interesting observation was that the ROI of the DL model seemed to differ slightly between right and left eyes. For right eye detection, the ROI mainly focused on the disc area, while for left eye detection, it was relatively bigger and located in the middle of the disc and macular region. This could be more obviously noticed based on left and right eye fundus images from the same person (Fig 6).

## Discussion

In this study we developed a self-adaptive DL model for eye laterality detection based on fundus color images with real-world labels. Even though current digital fundus cameras can automatically insert eye laterality according to the image capture sequence, it is not feasible when trying to analyze a large quantity of fundus images from different cameras. Our DL model could be directly used in clinical practice for fundus images with different pathologies and by different cameras, and could also be useful for large open-access datasets without previous eye laterality labels.

In the internal validation, our model achieved a sensitivity of 99.18% and a specificity of 99.08% in eye laterality detection, which to the best of our knowledge, is higher than previously reported models in eye laterality detection. The algorithm was initially developed from a dataset collected in a population-based study using one single fundus camera, but then validated and proved to have superior accuracy in an independent image dataset where the images were collected with multiple fundus cameras from various clinical settings. In addition, the big sample size, use of real-world labelling and a self-adaptive strategy, as well as comparison of multiple image preprocessing methods were all considered strengths of this study. Previously, Tan et al. proposed an image processing method based on optic cup segmentation using pixel intensity, which was easily affected by pixel noises introduced by poor image quality or pathological changes.[6] The model was trained based on 194 images and achieved an accuracy of 92.23%. Roy et al. proposed a fusion feature method which used a transfer learning based deep convolutional neural network (CNN) to extract the global feature of the fundus image, then fused the local anatomical features which were extracted by manually defined rules for final classification.[7] They trained the model using 5000 images and achieved an accuracy of 94.00%. Jang et al. trained a deep CNN model to detect the laterality of fundus images, and showed the visualization of their model activations by a guided CAM method.[19] The sample included 25911 images in total and their model had an overall accuracy of 98.98%. Detailed comparison of the relevant models could be found in S1 Table.

Given that the images in our training and internal validation dataset were labeled by real-world eye laterality records, the trained DL model was more reliable and had more pragmatic value in clinical settings. To verify the real-world reliability of our model, we conducted an external validation in a real-world LabelMe Dataset. The model achieved a comparable result as in the internal validation. Only 19 out of 2000 images were misclassified, among which 12 images were further proved to be wrongly labeled by human upon verification by a external ophthalmologist, which emphasizes the importance of using real-world labelling in clinical DL applications. The remaining 7 images did not have a visualised optic disc, indicating the importance of the optic disc feature in DL detection of eye laterality based on fundus images. We also investigated the model performance in images with fundus pathologies. The final model could make correct decision with high confidence regardless of eye abnormality, as long as the major anatomical structures were visible. The ROI was always focused on the optic disc and its surrounding area, this ROI-invariant feature is crucial for human understanding when considering the future real-world application of our model. Moreover, it indicates that eye laterality meta information could be well removed by modifying or sheltering the optic disc area for privacy preserving need in future studies and data centers.

Preprocessing using denoising or enhancement algorithms is a common method in fundus color image processing, including the automated eye disease diagnosis or semantic segmentation based on fundus images.[10, 20] However, to the best of our knowledge, few studies have reported or compared the performance of different preprocessing methods. We compared 4 preprocessing methods based on 2000 images and found that CLAHE had the best performance in this study. This indicates that the color contrast information plays an important role in the eye laterality detection, and preprocessing methods should be carefully considered in fundus image-based detection or diagnosis using DL. For instance, for detection of lesions which rely on color information (e.g. hemorrhage detection), enhancing the color contract and brightness may lead to a better model performance. The preprocessing method could be embedded as a specific layer into the DL network in our study, thus an integrated, end-to-end and fully-automated solution is achieved and could be applied directly in clinical practice.

When a DL network gets trained excessively in an invariable training dataset, it may learn the underlying patterns of the training data as much as possible, and recognize them as the targeted patterns of the whole sample space, leading to overfitting.[21] Overfitting could result in a final model with poor generalization capacity, which performs well only in the training dataset, but fails to make proper prediction in other unseen datasets. In this study, both data augmentation and the proposed self-adaptive strategy were performed to avoid overfitting. Learning rate, which stands for the speed of gradient descent in back-propagation, has a significant influence in DL network training.[22] Specifically, a too big learning rate would compromise the solving accuracy, while a too small rate could lead to local optimization rather than a globally optimal solution. One strategy commonly used in previous studies to select a suitable learning rate is repeated trials with different value-fixed learning rate,[7, 20] which is time-consuming and cannot perform real-time adjustment when overfitting occurs. The self-adaptive strategy we proposed was able to automatically adjust the learning rate during model training and iterate based on the current best model. This strategy had proven effectiveness when compared to the non self-adaptive model in improving the training performance and minimizing the risk of overfitting.

The limitations of the current study include 1) fundus images were mainly collected from Chinese participants, thus necessitating the need for further validation of the performance of the proposed DL model in other ethnic groups. 2) our study only used fundus images with a 45 degree of view; the performance on other fundus images with larger or smaller views were unknown. 3) We found that the ROI for left and right eye detection differed in size and location, one possible explanation could be that the DL model mainly recognizes the related location and color intensity between the optic disc and macular, but ignores the inner symmetry. This finding still needs further validation and investigation.

In summary, we proposed a self-adaptive DL model with a high performance for eye laterality detection based on fundus images with real-world labels, the necessity of image preprocessing selection and the importance of using real-world labels in DL model training were also addressed.

## Supporting information

S1 Fig Three different sizes of original images. (a) 2560*1920; (b) 3280*2480; (c) 4700*3100.
(PNG)

S2 Fig Image size normalization process. (a) the rescaled image with a size of 299*299; (b) circular mask for filtering the overexposed edges; (c) extracted key fundus area.
(PNG)

S3 Fig The fine-tuned Inception-V3 network for eye laterality detection. This network consists of 11 inception modules.
(PNG)

S4 Fig Receiver operating characteristic curve of the deep learning model for left eye detection in the external dataset. (PNG)

S5 Fig The seven poor quality images which were wrongly labeled by the deep learning model. (PNG)

S1 Table Details of the related studies on eye laterality detection. (DOCX)
