# Supplementary figures and images for: A self-adaptive deep learning method for automated eye laterality detection based on color fundus photography
Source: PLoS One. 2019 Sep 19;14(9):e0222025. doi: 10.1371/journal.pone.0222025 (PMC6752776; doi:10.1371/journal.pone.0222025)

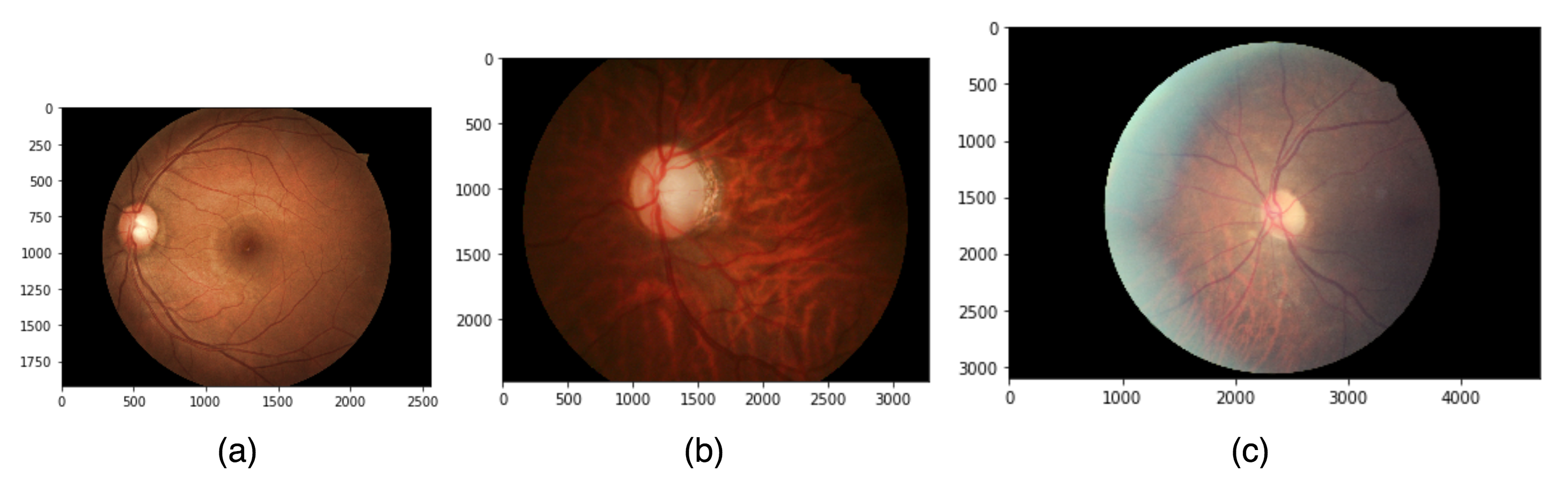

Supplement: S1 Fig — (a) 2560*1920; (b) 3280*2480; (c) 4700*3100. (PNG) [file pone.0222025.s001.png]

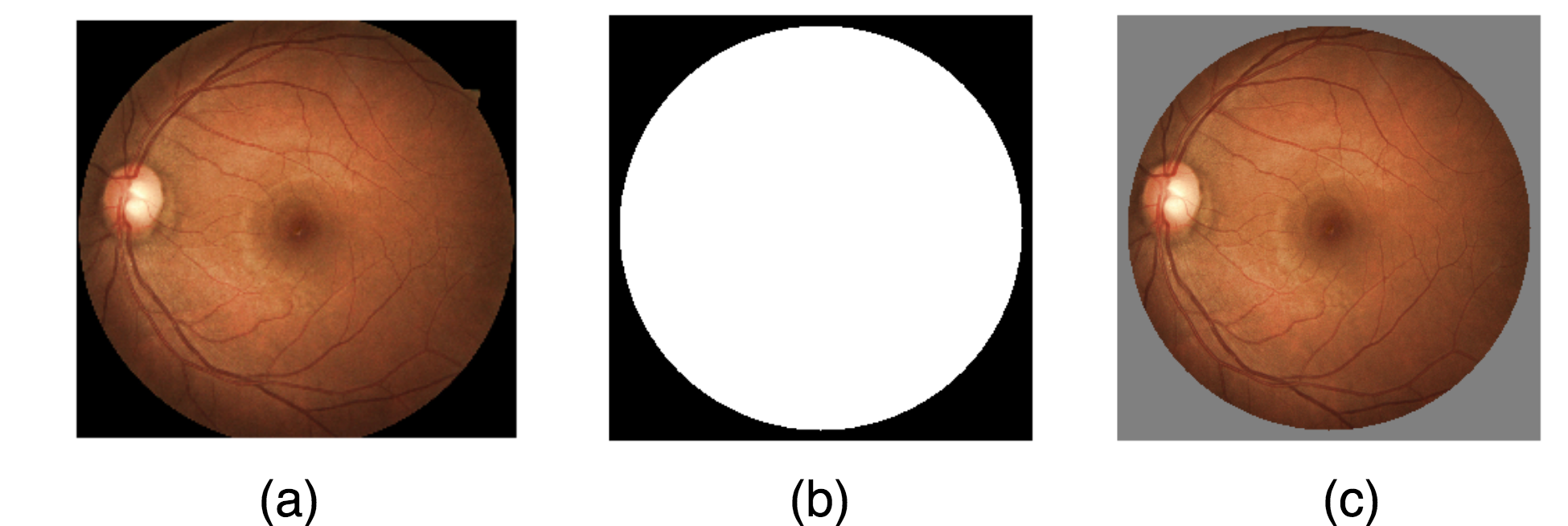

Supplement: S2 Fig — (a) the rescaled image with a size of 299*299; (b) circular mask for filtering the overexposed edges; (c) extracted key fundus area. (PNG) [file pone.0222025.s002.png]

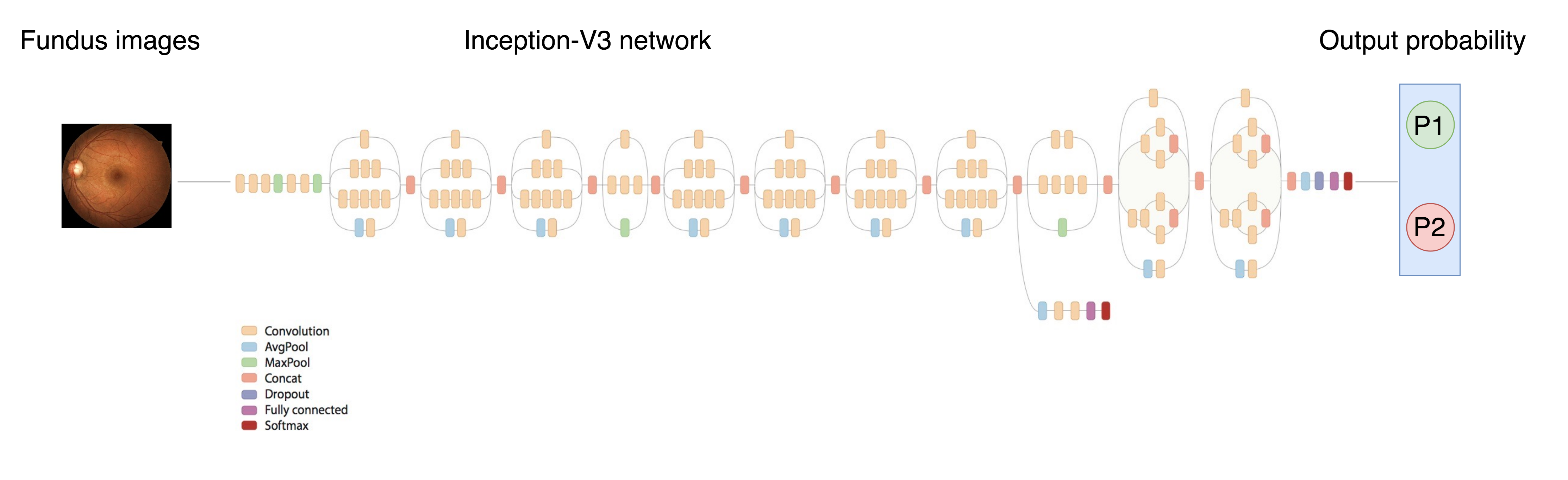

Supplement: S3 Fig — This network consists of 11 inception modules. (PNG) [file pone.0222025.s003.png]

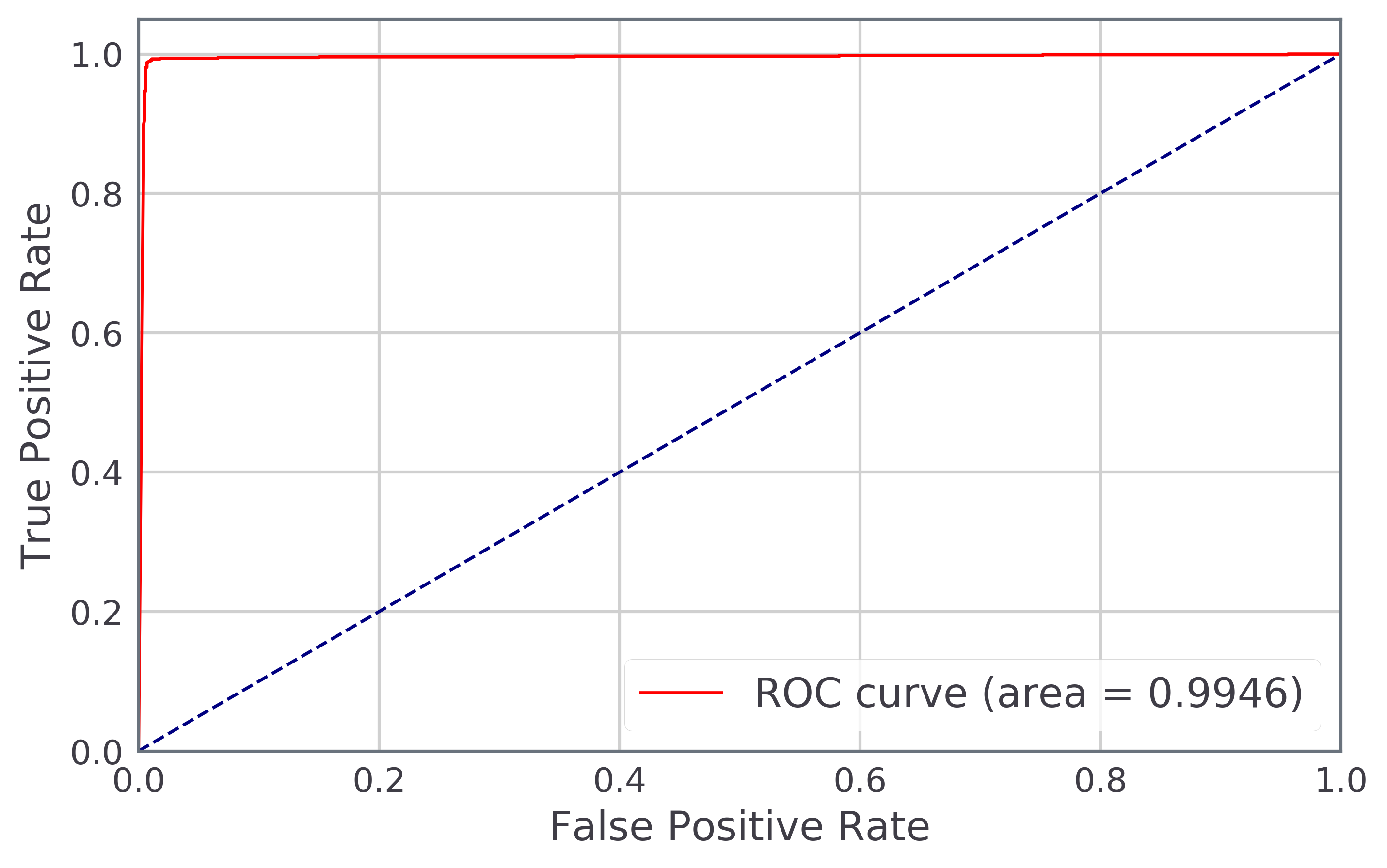

Supplement: S4 Fig — (PNG) [file pone.0222025.s004.png]

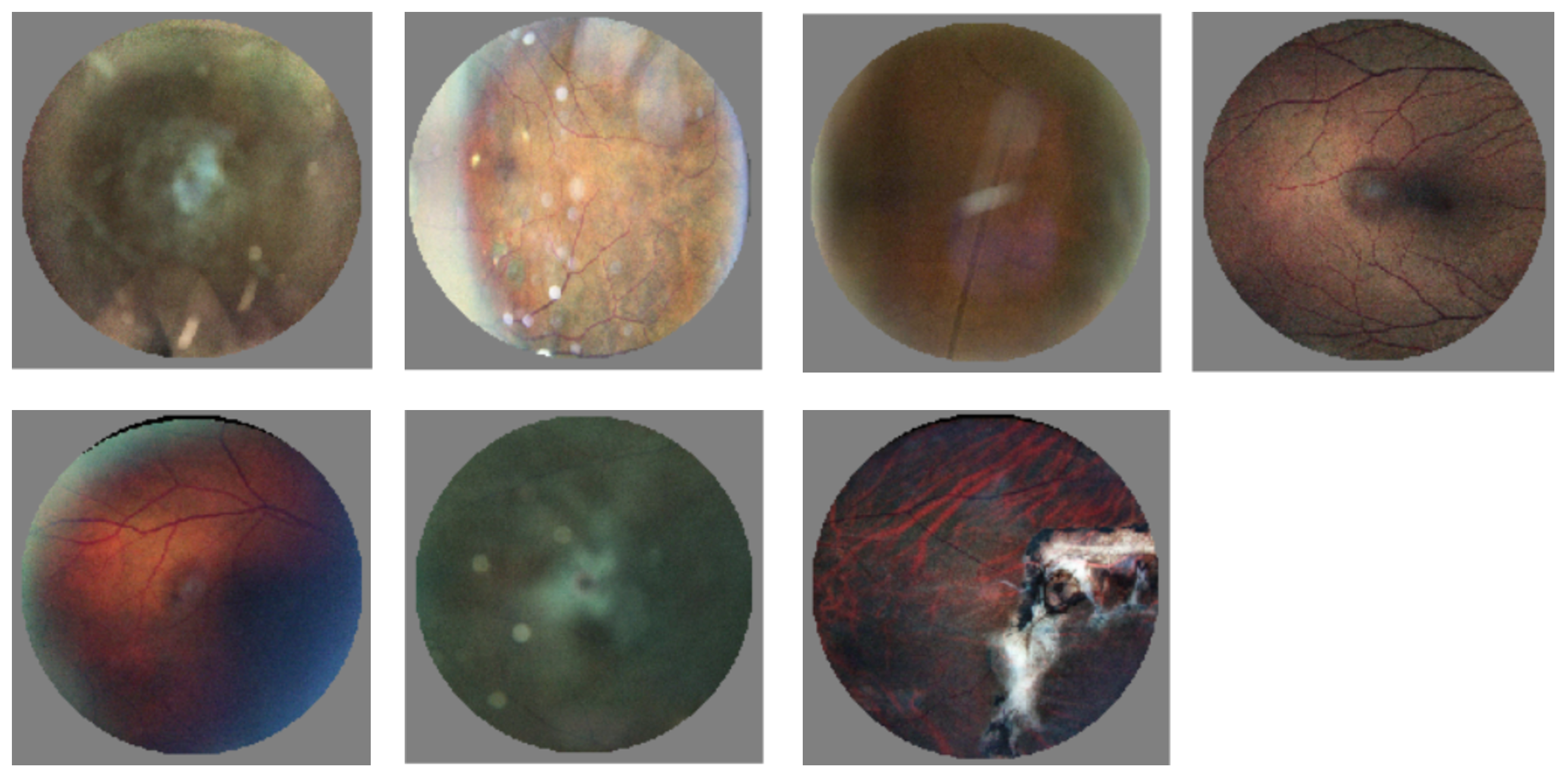

Supplement: S5 Fig — (PNG) [file pone.0222025.s005.png]
