# Supplementary material for: A self-adaptive deep learning method for automated eye laterality detection based on color fundus photography
Source: PLoS One. 2019 Sep 19;14(9):e0222025. doi: 10.1371/journal.pone.0222025 (PMC6752776; doi:10.1371/journal.pone.0222025)
Supplement: S1 Table — (DOCX) [file pone.0222025.s006.docx]

S1 Table. Details of the related studies on eye laterality detection

|  | Dataset size | Multi-centres study | Accuracy | AUC |
| --- | --- | --- | --- | --- |
| Tan et al.^6^ | 194 | No | 92.23% | - |
| P. K. Roy et al.^7^ | 5000 (Training 60%,  internal validation 40%) | Yes | 94.00% | 0.990 |
| Jang et al.^19^ | 25,911 (Training 80%,  internal validation 20%) | No | 98.98% | - |
| Our method | 18394 (Training 80%,  internal validation 20%)  External validation 2000 | Yes | 99.02% | 0.9946 |
